# Predictive Value of Immune Cells in the Risk of Gestational Diabetes Mellitus: A Pilot Study

**DOI:** 10.3389/fcdhc.2022.819164

**Published:** 2022-02-18

**Authors:** Adnette Fagninou, Magloire Pandoua Nekoua, Salomon Ezéchiel M. Fiogbe, Kabirou Moutaïrou, Akadiri Yessoufou

**Affiliations:** ^1^ Laboratory of Cell Biology, Physiology and Immunology, Department of Biochemistry and Cellular Biology, Faculty of Sciences and Technology (FAST), Institute of Applied Biomedical Sciences (ISBA), University of Abomey-Calavi (UAC), Cotonou, Benin; ^2^ Unité de Recherche sur les Maladies Non Transmissibles et le Cancer (UR-MNTC), Laboratory of Research in Applied Biology (LARBA), Ecole Polytechnique d’Abomey-Calavi, University of Abomey-Calavi, Cotonou, Benin

**Keywords:** immune cells, biochemical parameters, gestational diabetes mellitus, predictive value, lymphocyte, HDL-cholesterol

## Abstract

**Aims:**

Immunological and biochemical parameters are gaining more and more importance in the prognosis of diabetes and its complications. Here, we assessed the predictive power of immune cells correlated with biochemical parameters in gestational diabetes mellitus (GDM).

**Materials and Methods:**

Immune cells and serum biochemical parameters were determined in women with GDM and pregnant controls. Receiver operating characteristics (ROC) curve analyses were conducted to assess the optimal cutoff and value of ratios of immune cells to biochemical parameters for predicting GDM.

**Results:**

Blood glucose, total cholesterol, LDL-cholesterol and triglycerides were significantly increased whereas HDL-cholesterol decreased in women with GDM compared to pregnant controls. Glycated hemoglobin, creatinine, transaminase activities did not significantly differ between both groups. Total leukocyte, lymphocyte and platelet numbers were significantly high in women with GDM. Correlation tests showed that ratios of lymphocyte/HDL-C, monocyte/HDL-C and granulocyte/HDL-C were significantly higher in women with GDM than in pregnant controls (*p* = 0.001; *p* = 0.009 and *p* = 0.004 respectively). Women with a lymphocyte/HDL-C ratio greater than 3.66 had a 4-fold increased risk of developing GDM than those with lower ratios (odds ratio 4.00; 95% CI: 1.094 – 14.630; *p*=0.041).

**Conclusion:**

Our study showed that ratios of lymphocyte, monocyte and granulocyte to HDL-C might represent valuable biomarkers for GDM and in particular, lymphocyte/HDL-C ratio exhibited a strong predictive power for GDM risk.

## Introduction

One of the major concerns of researchers is to find biological or clinical factors with prognostic or early diagnostic value of diseases in order to strengthen or improve prevention rather than cure. In this context, little is known about the use of immunological and/or biochemical parameters in the prediction of gestational diabetes mellitus (GDM) (1, 2). Recently, we investigated the modulation of immune cell frequencies in gestational diabetes, and found that gestational diabetes mellitus (GDM) modulated the frequencies of total CD3+ and CD4+ T and B cells, suggesting that immune cells could play specific role in the prognosis of this disease (3). GDM is defined as glucose intolerance arising for the first time during pregnancy with or without remission after the end of pregnancy (4, 5). GDM, as one of major endocrine abnormalities, is the most common metabolic disease during pregnancy and its incidence is increasing worldwide (4, 5). The global prevalence of GDM varies from 1 to 28% depending on population characteristics, screening methods, and diagnostic criteria (6–8) with a great percentage reported in low and middle-income countries, where access to maternal care is often limited (9). Sedentary and modern lifestyle in developing countries contribute to the increased prevalence of GDM (10, 11).

Evidently, immunological parameters including immune cell subpopulations and cytokines have been designated as predictors of endothelial dysfunction and inflammation (12). Likewise, we have recently reported that immune cell frequencies, including neutrophils, eosinophils, monocytes, NK cells, and lymphocytes, can be modulated in type 1 diabetes and type 2 diabetes whether associated with pregnancy or not, suggesting that these cells can play important roles in the pathogenesis of this disease, on the one hand (3, 13, 14). On the other hand, we have reported that GDM can induce disruption of several biochemical and immunological parameters (3, 15, 16). Additionally, we have reported that biochemical parameters, including glycaemia, triglycerides (TG), high density lipoprotein-cholesterol (HDL-C), total cholesterol (TC), low density lipoprotein-cholesterol (LDL-C), known as metabolic biomarkers, are modulated during GDM and macrosomia (15, 17–20). Interestingly in the same way, several studies have found that immune parameters, including lymphocytes, neutrophils, monocytes, platelets, and the ratios between these cells and HDL-C, may be related to metabolic syndrome and atherosclerotic processes, as potential indicators of prothrombotic and pro-inflammatory states (21–24). Consequently, early diagnosis of gestational diabetes, based on biochemical and immunological parameters, could be crucial to anticipate the care of pregnant diabetic women and thus, prevent the wide range of adverse consequences on the offspring, including macrosomia, fetal death, prematurity, birth trauma, respiratory distress syndrome, obesity, impaired glucose tolerance, and type 2 diabetes in adulthood (15, 20). Evidently, biochemical parameters can be easily determined in plasma and immune parameters can be easily measured from peripheral blood. Biochemical and immunological indicators, as discussed above, can be used as potential markers to predict GDM. Therefore, the principal objective of this study is to determine whether immune cells could be correlated with biochemical parameters to assess their predictive value for GDM.

## Materials and Methods

### Study Participants

In this cross-sectional and descriptive study, two hundred and forty-six (246) pregnant women were firstly enrolled by specialist clinicians of the department of gynecology and obstetrics three national hospital centers in southern Benin. This sample size was calculated based on Dagnelie’s formula. Based on inclusion criteria including absence of preexisting type 1 or type 2 diabetes, infectious diseases including hepatitis, HIV and malaria after blood sample tests, 210 pregnant women, aged from 19 to 43 years, were selected and then screened for GDM (see protocol below). Anthropometric and socio-demographic data, risk factors and family history associated with diabetes were recorded and presented in **Table 1**.

**Table 1:** Anthropometric data of subjects.

Characteristics	Pregnant control women		Women with GDM		Total
	Number	Percentage (%)	Number	Percentage (%)	
**Number of subjects**	185	88.10	25	11.90	210
**Age (A, years)**					
< 20	05	2.70	00	00	05
20 ≤ A < 30	104	56.22	09	36	113
30 ≤ A < 40	71	38.38	15	60	86
≥ 40	05	2.7	01	04	06
**Physical activity**					
Intense	00	00	00	00	00
Moderate	136	73.51	17	68	153
Inadequate	49	26.49	08	32	57
**Menstrual cycle**					
Regular	80	43.24	10	40	90
Irregular	105	56.76	15	60	120
**Number of children (N)**					
0	50	27.02	02	08	52
1 ≤ N ≤ 2	97	52.44	07	28	104
N ≥ 3	38	20.54	16	64	54
**Previous disturbances**					
Miscarriage	44	23.78	12	48	56
Prematurity	07	3.78	04	16	11
Normal delivery	134	72.43	09	36	143

Gestational diabetes mellitus (GDM) was diagnosed in pregnant women following the protocol of International Association of Diabetes and Pregnancy Study Group (IADPSG). Using overnight fasting glucose and OGTT test in pregnant women between 24 and 28 weeks of gestation Subjects were declared as positive for GDM positive when overnight fasting plasma glucose was ≥ 92 mg/dL (5.1 mmol/L), or 1-hour OGTT plasma glucose level was ≥ 180 mg/dL (10.0 mmol/L), or 2-hours OGTT plasma glucose level was ≥ 153 mg/dL (8.5 mmol/L). Moderate physical activity = 30 minutes of physical activity per day; Inadequate physical activity = insufficient or no physical activity. Regular menstrual cycle: time between successive menstruations is relatively regular and predictable. Irregular menstrual cycle: time between successive menstruations is very variable and unpredictable.

The study was conducted in accordance with the Declaration of Helsinki 1964 (as revised in Edinburgh 2000) and was approved by the Ethics Committee on Research of the Institute of Applied Biomedical Sciences of Cotonou, Benin under the number Dec.n°100/CER/ISBA-2016. Prior to enrollment, written consent was obtained from each participant who were informed of the study aim. The privacy rights of human subjects were observed.

### Screening of Gestational Diabetes Mellitus

Gestational diabetes mellitus was diagnosed in pregnant women following the protocol of the International Association of Diabetes and Pregnancy Study Group (IADPSG) (25). Briefly, women between 24 and 28 weeks of gestation after overnight fasting were submitted to an oral glucose tolerance test (OGTT**)** and given 75 grams of glucose. Subjects were declared as positive for GDM when overnight fasting plasma glucose was ≥ 92 mg/dL (5.1 mmol/L), or 1-hour OGTT plasma glucose level was ≥ 180 mg/dL (10.0 mmol/L), or 2-hours OGTT plasma glucose level was ≥ 153 mg/dL (8.5 mmol/L).

The GDM screening revealed that 25 pregnant women have gestational diabetes, representing a percentage of 11.90%, and considered as the cases’ group. Pairing of these 25 newly GDM diagnosed women with non-diabetic pregnant women, according to age, body mass index and gestational age, allowed us to select 35 pregnant women without GDM, and considered as control group. Therefore, both groups of participants, twenty-five women with GDM and thirty-five age-matched and body mass index-matched and gestational age-matched pregnant controls were selected and submitted for blood collection and biochemical and immunological assays.

### Blood Samples

Blood samples were collected from each selected participant in appropriate tubes and immediately transported to the laboratory for biological assays within 2 hours. Immune parameters and glycated hemoglobin (HbA1c) were determined in whole blood. Plasma samples were immediately used for glucose determination. Serum obtained by low-speed centrifugation was used for biochemical assays.

### Biochemical Assays

Plasma glucose, total cholesterol, HDL cholesterol, triglycerides were measured by colorimetric enzymatic method using ELITech reagents (ELITech Group, Puteaux, France) according to manufacturer’s instructions. LDL-cholesterol was calculated using Friedewald method (26). Total protein levels were determined by direct Biuret colorimetric method (ELITech Group, Puteaux, France). Aspartate aminotransferase (AST) and alanine aminotransferase (ALT) enzymatic activities and creatinine levels were determined by enzymatic kinetic assay (DiaSys reagents, Diagnostic Système GmbH, Germany). HbA1c concentration was calculated using a percentage of total hemoglobin, according to the manufacturer’s instructions (Reference 41190, Labkit Chemelex SA, Barcelona, Spain).

### Determination of Immune Cells

Immune cells were determined through the complete blood formula count using an automatic blood cell analyzer (Cell Dyn 3500, Abbott, France). These cells included total leukocytes, lymphocytes, monocytes, granulocytes and platelets (PLT).

### Statistical Analysis

Data analyses were performed using Graph Pad Prism 6.0 (Graph Pad Inc., CA, USA) and IBM^®^ SPSS^®^ Statistics (version 25.0). Values are means ± standard deviation or medians with interquartile ranges. Student’s t-test, Mann–Whitney U test and Chi-squared (χ2) test were used when appropriate. Pearson and Spearman correlations were used to determine the association between immunological and biochemical parameters. Receiver Operating Characteristics (ROC) curve analysis was used to assess the value of immunological to biochemical parameter ratios for predicting gestational diabetes mellitus and to obtain the best cutoff value using Youden’s index (sensitivity + specificity – 1). The odds ratios (ORs) are presented with 95% confidence intervals (CI). Differences were considered significant with a two-tailed *p* value < 0.05.

## Results

### Biochemical Parameters in Women With GDM and Pregnant Controls

Biochemical parameters of women with GDM and pregnant controls are presented in **Table 2**. We observed that plasma fasting glucose (*p* < 0.001), total cholesterol (*p* = 0.001), LDL cholesterol (*p* = 0.015), triglyceride (*p* = 0.035) and total protein (*p* = 0.005) and HDL cholesterol (*p* = 0.001) levels significantly increased, while HDL-cholesterol level decreased in women with GDM compared to pregnant controls (**Table 2**). However, HbA1c and creatinine levels, and transaminase (AST and ALT) activities did not significantly differ between the two groups of women (**Table 2**).

**Table 2:** Biochemical parameters in women with GDM and pregnant controls.

Parameters	Pregnant control women (n = 35)	Women with GDM (n = 25)	p-value
**Glucose (g/L)**	0.81 ± 0.03	1.16 ± 0.04	0.001
**HbA1c (%)**	5.65 ± 0.23	6.47 ± 0.48	0.451
**TC (g/L)**	1.53 ± 0.11	2.11 ± 0.31	0.001
**HDL-C (g/L)**	1.21 ± 0.15	0.35 ± 0.10	0.001
**LDL-C (g/L)**	0.91 (0.14-1.36)	1.53 (1.15-1.68)	0.015
**TG (g/L)**	1.30 ± 0.08	1.63 ± 0.18	0.035
**AST (UI/L)**	26.63 ± 2.64	26.20 ± 6.46	0.951
**ALT (UI/L)**	10.50 (9.75-15.25)	14.00 (12.00-20.00)	0.425
**Creatinine (mg/L)**	7.30 ± 0.45	8.92 ± 0.30	0.125
**Total proteins (g/L)**	71.60 ± 1.585	82.23 ± 3.32	0.005

TC, Total cholesterol; HDL-C, HDL cholesterol; LDL-C, LDL cholesterol; TG, triglycerides. Friedewald method was used to calculate LDL-cholesterol. LDL-C = CT – (HDL-C+TG/5) in g/l. This method is valid as the TG levels were under 4 g/l in the present study. Aspartate aminotransferase (ASAT); Alanine aminotransferase (ALAT). Statistical analyses were performed using the Student’s t-test or Mann-Whitney test. p values < 0.05 indicate significant differences. n = 25 women with GDM, n= 35 pregnant women without GDM as control group.

### Immune Cells in Women With GDM and Pregnant Controls

**Figure 1:**
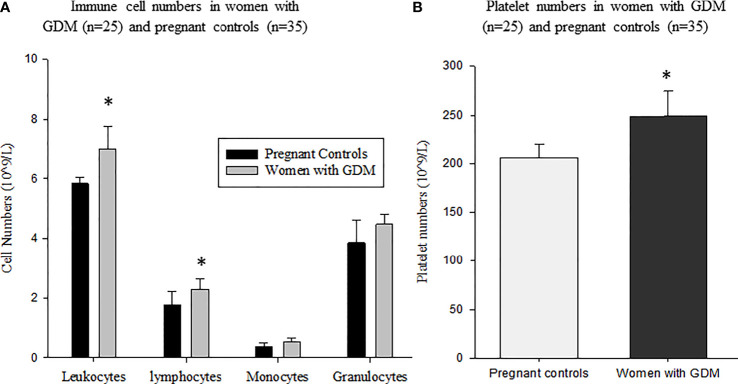
Immune cell numbers in women with GDM (n = 25) and pregnant women without GDM as control group (n = 35): **(A)** total leukocytes, lymphocytes, monocytes and granulocytes; **(B)** Platelet numbers in women with GDM and pregnant controls. Values are means ± SD. *p values (*p* < 0.05) indicate significant difference between women with GDM and pregnant controls. Statistical analyses were performed using the Student’s t-test or Mann-Whitney test.

**Figure 2:**
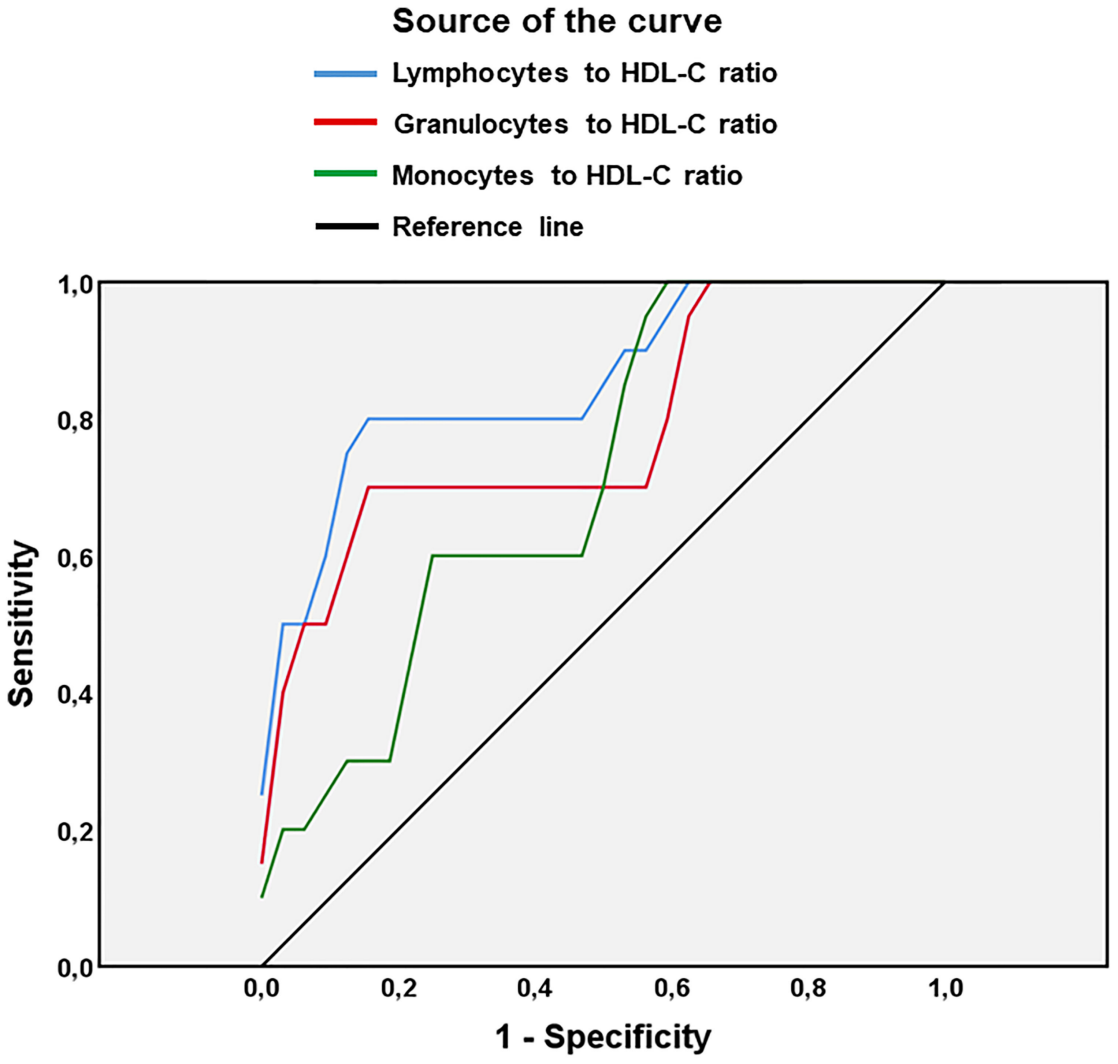
Receiver Operating Characteristics (ROC) curve analysis of the value of ratios lymphocytes/HDL-C, granulocytes/HDL-C and monocytes/HDL-C for predicting gestational diabetes mellitus in pregnant women. HDL-C, high-density lipoprotein - cholesterol. n = 25 women with GDM, n= 35 pregnant women without GDM as control group.

Immune cell percentages of women with GDM compared to pregnant controls are shown in **Figure 1**. Total leukocytes (*p* = 0.045), lymphocytes (*p* = 0.015) and platelet numbers (*p* = 0.033) were significantly higher in women with GDM than in pregnant controls. However, no significant difference was observed in the numbers of monocytes and granulocytes between both groups (**Figures 1**, **2**).

### Correlation Between Immune Cells and Biochemical Parameters

In pregnant control women, the correlation tests revealed a positive correlation between blood glucose with lymphocytes (r = 0.89; *p* = 0.03) and between lymphocytes with total cholesterol (r = 0.50; *p* = 0.04) (**Table 4**). Moreover, a positive correlation was found between monocytes with triglycerides (r = 0.58; *p* = 0.04). In contrast, a negative correlation was observed between monocytes and HDL-cholesterol levels (r = − 0.68; *p* = 0.007) (**Table 3**).

**Table 3:** Correlations between immune cell subpopulations and biochemical parameters in pregnant control women (n = 35).

Immune cell subtypes	Glucose		TC		HDL-C		LDL-C		TG	
	*r*	p	*r*	p	*r*	p	*r*	p	*r*	p
Leucocytes	-0.50	0.45	0.37	0.22	-0.19	0.46	-0.42	0.09	0.37	0.24
Lymphocytes	0.89	0.03*	0.50	0.04*	0.36	0.17	0.11	0.68	0.16	0.73
Monocytes	-0.45	0.40	-0.43	0.08	-0.68	0.007*	0.36	0.17	0.58	0.04*
Granulocytes	-0.81	0.07	-0.27	0.30	0.22	0.39	-0.25	0.35	-027	0.39
Platelets	0.68	0.08	-0.33	0.20	-0.36	0.18	0.20	0.45	0.33	0.27

TC, Total cholesterol; HDL-C, HDL cholesterol; LDL-C, LDL cholesterol; TG, triglycerides. Spearman or Pearson correlation tests were used when appropriate. *p values < 0.05 indicate significant differences.

In women with GDM, there was a positive correlation between glucose with leukocytes (*r* = 0.70; *p* = 0.03) on the one hand and between glucose with lymphocytes (*r* = 0.67; *p* = 0.02) on the other hand (**Table 4**). Also, we noticed a positive correlation between serum triglycerides with monocytes (*r* = 0.87; *p* = 0.045). In contrast, a negative correlation between granulocytes with HDL cholesterol was noted (*r* = − 0.90; *p* = 0.026).

**Table 4:** Correlations between immune cell subpopulations and biochemical parameters in women with GDM (n = 25).

Immune cell subtypes	Glucose		TC		HDL-C		LDL-C		TG	
	*r*	p	*r*	p	*r*	p	*r*	p	*r*	p
Leucocytes	0.7	0.03*	0.20	0.76	0.66	0.26	0.61	0.30	0.21	0.76
Lymphocytes	0.6	0.02*	0.12	0.99	0.81	0.13	-0.12	0.95	0.16	0.95
Monocytes	-0.23	0.66	0.66	0.28	0.37	0.15	0.66	0.26	0.87	0.04*
Granulocytes	0.72	0.23	-0.48	0.5	-0.9	0.02*	-0.41	0.51	-0.2	0.78
Platelets	-0.51	0.45	0.73	0.23	0.8	0.13	0.70	0.23	0.11	0.97

TC, Total cholesterol; HDL-C, HDL cholesterol; LDL-C, LDL cholestero; TG, triglycerides. Spearman or Pearson correlation tests were used when appropriate. *p values < 0.05 indicate significant differences.

### Ratios of Immune Cells to Biochemical Parameters for Predicting GDM

**Table 5** shows ratios between immunological to biochemical parameters in women with GDM and pregnant controls. We found that ratios of lymphocytes/HDL-C, monocytes/HDL-C and granulocytes/HDL-C were significantly higher in women with GDM than in pregnant controls (*p* = 0.001; *p* = 0.009 and *p* = 0.004 respectively).

**Table 5:** Ratios between immune cells to biochemical parameters in women with GDM (n = 25) and pregnant control women (n = 35).

Variables	Pregnant control women (n = 35)	Women with GDM (n = 25)	*p*-value
Lymphocytes to Glucose ratio	2.48 ± 0.43	2.03 ± 0.59	0.125
Lymphocytes to HDL-C ratio	1.65 ± 0.86	7.38 ± 3.21	0.001*
Lymphocytes to LDL-C ratio	2.53 ± 0.58	1.62 ± 0.22	0.591
Lymphocytes to TG ratio	1.50 ± 0.27	1.25 ± 0.60	0.469
Monocytes to Glucose ratio	0.34 ± 0.09	0.47 ± 0.13	0.424
Monocytes to HDL-C ratio	0.39 ± 0.05	1.73 ± 0.49	0.009*
Monocytes to LDL-C ratio	0.41 ± 0.27	0.39 ± 0.15	0.701
Monocytes to TG ratio	0.25 ± 0.07	0.34 ± 0.09	0.117
Granulocytes to Glucose ratio	5.13 ± 1.24	3.33 ± 0.73	0.082
Granulocytes to HDL-C ratio	3.55 ± 2.02	14.18 ± 5.70	0.004*
Granulocytes to LDL-C ratio	5.16 ± 3.42	3.27 ± 0.95	0.657
Granulocytes to TG ratio	3.17 ± 0.61	2.91 ± 0.73	0.229

TC, Total cholesterol; HDL-C, HDL cholesterol; LDL-C, LDL cholestero; TG, triglycerides. Spearman or Pearson correlation tests were used when appropriate. *p values < 0.05 indicate significant differences.

As shown in **Figure 2**, a ROC curve analysis was used to assess the accuracy, sensitivity, specificity and value of the ratios of lymphocytes/HDL-C, granulocytes/HDL-C and monocytes/HDL-C for predicting GDM. The analysis showed that the lymphocytes/HDL-C ratio had a higher accuracy in predicting gestational diabetes mellitus (AUC = 0.859; *p* < 0.001; 95% CI: 0.752 - 0.966) than the granulocytes/HDL-C ratio (AUC = 0.787; *p* < 0.01; 95% CI: 0.654 -0.921) or the monocytes/HDL-C ratio (AUC = 0.716; *p* < 0.01; 95% CI: 0.576 - 0.855) (**Table 6**). The optimal cutoff values of lymphocytes/HDL-C ratio, granulocytes/HDL-C ratio and monocytes/HDL-C ratio for predicting GDM were, respectively, 3.66 (sensitivity = 80.0%; specificity = 50.1%); 5.50 (sensitivity = 70.3%; specificity = 59.4%) and 1.56 (sensitivity = 60.9%; specificity = 50.0%) (**Table 6**). Odds ratios were used to assess the risk of GDM. We observed that pregnant women with the lymphocytes/HDL-C ratio greater than 3.66 had a 4-fold increased risk of developing GDM than those with lower ratios (odds ratio 4.00; 95% CI: 1.094 – 14.630; *p* = 0.041) (**Table 7**).

**Table 6:** Areas Under the ROC Curve (AUC), Sensitivity and Specificity in predicting GDM.

Risk factor	AUC (95% CI)	Cutoff According to Youden’s Index	Sensitivity (%)	Specificity (%)
**Lymphocytes to HDL-C ratio**	0.859 (0.752-0.966)	3.66	80.0	50.1
**Granulocytes to HDL-C ratio**	0.787 (0.654-0.921)	5.50	70.3	59.4
**Monocytes to HDL-C ratio**	0.716 (0.576-0.855)	1.56	60.9	50.0

Areas Under the ROC Curve (AUC), Sensitivity and Specificity by the Optimized Cutoff Points for lymphocytes to HDL-C ratio, granulocytes to HDL-C ratio and monocytes to HDL-C ratio in predicting GDM. ROC, Receiver operating characteristics; AUC, Area under ROC curve; CI, Confidence interval.

**Table 7:** Odds ratio (OR) of independent predictors accessing risk of gestational diabetes mellitus in women with GDM (n = 25) and pregnant control women (n = 35).

Variable	OR (95% CI)	*p*-value
Lymphocytes to HDL-C ratio	4.000 (1.094 – 14.630)	0.041
Granulocytes to HDL-C ratio	1.596 (0.486 to 5.241)	0.557
Monocytes to HDL-C ratio	1.500 (0.483 – 4.652)	0.572

## Discussion

Increasingly, the identification of biological parameters that can facilitate the prediction and early prognosis of gestational diabetes mellitus (GDM) has become a major concern for researchers. Given the complications associated with GDM in mothers, fetuses, newborns and adult offspring, an early diagnosis of GDM could help anticipate the care of pregnant women and limit the adverse effects. Therefore, the aim of this study was to investigate whether immunological parameters like immune cells, in conjunction with biochemical parameters, could be used to predict the risk of GDM.

As far as metabolic aspect is concerned, diabetes is known to be associated with biochemical and metabolic disturbance (27). In the present study, we observed that HbA1C levels were normal and did not significantly differ between women with GDM and pregnant controls, although glycaemia remained high in women with GDM. The normal level of HbA1c might suggest that women with GDM were under an adequate metabolic control (28, 29). However, the fact that their glycemia remained high could suggest that women with GDM had poor glycemia control despite their normal HbA1c levels. In fact, it’s important to note that pregnancy can impact HbA1c levels independently of glycemia. In a study conducted in pregnant women without GDM, it has been reported that the HbA1c level was low in early pregnancy and even more reduced at the end of pregnancy compared to non-pregnant women of the same age, suggesting that the pregnancy can significantly influence HbA1c levels regardless of glycemia (17). In addition, this dichotomy could also be explained by the fact that women with GDM are newly diagnosed and they have not yet been subjected to any anti-diabetic treatment (28, 29).

It is commonly believed that GDM is associated with the modulation of lipid profiles. Although the results describing lipid profiles during normal pregnancy and GDM are diverse and extensive, the results have been inconsistent (15, 17–20, 30, 31). The present study showed that serum TC, LDL-C and TG levels increased significantly, while HDL-C levels decreased in women with GDM compared to pregnant controls. These results are in agreement with others who have also shown a significant decrease in HDL levels in pregnant women with glucose intolerance compared to control women (32, 33). However, other studies have noted a significant rise in all lipids including HDL-C in women with GDM from the middle of the 2nd trimester of pregnancy to reach their peak at childbirth (34). A meta-analysis showed that GDM was associated with elevated serum TG in the 3rd trimester of pregnancy, while serum HDL-C levels were significantly low in the 2^nd^ and 3^rd^ trimesters (30). Indeed, during normal pregnancy, circulating lipids markedly increase, due to estrogen stimulation and insulin resistance (35). High maternal fat accumulation during pregnancy has also been shown to be associated with both overeating and increased fetal lipogenesis and energy demand, necessary for childbirth and lactation (27, 36–38). In GDM, the situation appears to be similar as lipid levels increased during pregnancy. In fact, the increased levels of TG, TC, and LDL-C observed in GDM in the present study could lead to increased lipid storage in women with GDM, due to decreased lipolytic clearance of TG and increased hepatic lipase activity which appears to lead to increased HDL catabolism (39, 40).

As far as immunological aspects are concerned, there is evidence that gestational diabetes induces a profound variation of immune parameters (41). Indeed, we have recently demonstrated that GDM was associated with high frequencies of total CD3+ and CD4+ T lymphocytes and B cells, suggesting that GDM could induce a concomitant activation of cellular and humoral immunity (3). Likewise in the present study, we found that total leukocytes and lymphocytes in particular significantly increased in women with GDM as compared to control pregnant. There was no significant difference in granulocyte and monocyte numbers in both groups of women. These results are in agreement with previous work which reported that increased inflammatory cellular markers were associated with impaired glucose metabolism, insulin resistance and GDM (42–44). Evidently, the increased numbers of leukocytes and lymphocytes in women with GDM were consistent with the increase of a wide range of inflammatory metabolic markers such as TG, TC and LDL-cholesterol which together lead to insulin resistance (42–44). In addition, we noticed a significant increase in the number of platelets in women with GDM compared to control women. These results were similar to those of Lim et al. (23) who have shown that high platelet numbers was associated with an increased prevalence and risk of metabolic syndrome in children and adolescents.

All these observations prompted us to investigate the correlations between immunological and metabolic parameters during GDM. In fact, we observed, in both pregnant controls as well as in women with GDM, a positive correlation between blood glucose and total lymphocytes, between TG and monocytes; between TC and lymphocytes in pregnant controls and between blood glucose and total leukocytes in women with GDM on the one hand. On the other hand, we found a negative correlation between HDL-cholesterol and monocytes in pregnant controls and between HDL-cholesterol and granulocytes in women with GDM. All these correlations suggested that these parameters could be useful in predicting GDM.

In order to determine whether both parameters could help in the prediction of GDM, we evaluated the ratios between immune cells (lymphocytes, granulocytes and monocytes) and biochemical parameters (glucose, TC, TG and LDL-C). Interestingly, we found that lymphocytes/HDL-C, monocytes/HDL-C, and granulocytes/HDL-C ratios were significantly higher in women with GDM than in pregnant controls, suggesting that these ratios may certainly have significant value in predicting GDM. In fact, analysis of odds ratios indicated that only pregnant women with a lymphocytes/HDL-C ratio greater than 3.66 have a 4.0-fold higher risk of developing GDM than those with a lymphocyte-to-HDL-C ratio lower (odds ratio 4.00; 95% CI: 1,094 - 14,630; *p* = 0.041).

Moreover, we would like to highlight the role of HDL-C in the present results as this lipoprotein seems to represent a central parameter to which immune cell frequencies could be added to more reliably determine the pathogenesis of GDM. In fact, HDL-C, as an anti-atherogenic lipoprotein, is recognized as a protective factor in atherosclerosis and inflammation (45, 46). It has also been reported that TG/HDL-C ratio is a better marker for evaluating insulin resistance and diabetes (47). In addition, previous studies have shown that immune cells can be used as novel markers for predicting inflammation, metabolic syndromes, diabetes and atherosclerosis (48). Indeed, Pattanathaiyanon et al. (49) demonstrated that increased leucocyte numbers in early pregnancy may lead to a significant risk of GDM. Wolf et al. (50) have also previously reported that leucocyte numbers greater than 9100 cells/μL in early pregnancy were significantly associated with a heightened risk of GDM.

To the best of our knowledge, our study is the first which analyzes the predictive power of immuno-biochemical markers in GDM, through ratios of lymphocytes, monocytes and granulocytes and HDL-C levels. Among these markers, the lymphocytes/HDL-C ratio seems to have a strong predictive power in the onset and development of GDM, and these parameters are easily accessible in patients. Even though the sample size was relatively small in this study, the causative effect of immune-metabolic biomarkers in GDM needs to be more investigated by including, in addition to immune cells, other inflammatory markers such as cytokines and chemokines. This aspect could be addressed in future investigations.

## Conclusion

The present results constitute a major advance in the use of biological parameters for prediction of GDM. Immune cells associated with biochemical parameters appear as valuable markers which can allow to predict GDM. The interest of this study lies in the fact that these markers can be easily assessed on automatic devices which are usually found in medical analysis laboratories and that the interpretation of data is relatively simple. Pending future investigations that may involve other markers, we hope that the present results may be useful to clinicians and biologists specializing in the care of pregnant women.

## Data Availability Statement

The original contributions presented in the study are included in the article/Supplementary Material. Further inquiries can be directed to the corresponding author.

## Ethics Statement

The study was conducted in accordance with the Declaration of Helsinki 1964 (as revised in Edinburgh 2000) and was approved by the Ethics Committee on Research of the Institute of Applied Biomedical Sciences (CER-ISBA) of Cotonou, Benin under the number Dec.n°100/CER/ISBA-2016. Prior to enrollment, written consent was obtained from each participant who were informed of the study aim. The privacy rights of human subjects were observed. The patients/participants provided their written informed consent to participate in this study.

## Author Contributions

AF was in charge of major parts of technical aspects of work and participated in the manuscript writing. SF and MN participated in the technical work and participated in the interpretation of data. KM participated in the manuscript writing. AY designed the study, supervised the work, wrote the manuscript and established the collaborative aspects. All authors read and approved the final manuscript.

## Funding

This work did not receive any funding from any organization. The work was financed from the authors’ own funds. Products and reagents were purchased by the contribution of the authors themselves.

## Conflict of Interest

The authors declare that the research was conducted in the absence of any commercial or financial relationships that could be construed as a potential conflict of interest.

## Publisher’s Note

All claims expressed in this article are solely those of the authors and do not necessarily represent those of their affiliated organizations, or those of the publisher, the editors and the reviewers. Any product that may be evaluated in this article, or claim that may be made by its manufacturer, is not guaranteed or endorsed by the publisher.
